# Individual Optimization of the Insertion of a Preformed Cochlear Implant Electrode Array

**DOI:** 10.1155/2015/724703

**Published:** 2015-09-10

**Authors:** Thomas S. Rau, Thomas Lenarz, Omid Majdani

**Affiliations:** Department of Otolaryngology, Hannover Medical School, Carl-Neuberg-Straße 1, 30625 Hannover, Germany

## Abstract

*Purpose*. The aim of this study was to show that individual adjustment of the curling behaviour of a preformed cochlear implant (CI) electrode array to the patient-specific shape of the cochlea can improve the insertion process in terms of reduced risk of insertion trauma.* Methods*. Geometry and curling behaviour of preformed, commercially available electrode arrays were modelled. Additionally, the anatomy of each small, medium-sized, and large human cochlea was modelled to consider anatomical variations. Finally, using a custom-made simulation tool, three different insertion strategies (conventional Advanced Off-Stylet (AOS) insertion technique, an automated implementation of the AOS technique, and a manually optimized insertion process) were simulated and compared with respect to the risk of insertion-related trauma. The risk of trauma was evaluated using a newly developed “trauma risk” rating scale.* Results*. Using this simulation-based approach, it was shown that an individually optimized insertion procedure is advantageous compared with the AOS insertion technique.* Conclusion*. This finding leads to the conclusion that, in general, consideration of the specific curling behaviour of a CI electrode array is beneficial in terms of less traumatic insertion. Therefore, these results highlight an entirely novel aspect of clinical application of preformed perimodiolar electrode arrays in general.

## 1. Introduction

An essential, but also risky, step in cochlear implantation is the insertion of the electrode array (EA) of a cochlear implant into the helically shaped scala tympani. The scala tympani is one of three tubular lumens inside the auditory portion (cochlea) of the inner ear. At one side, it is bordered by a fragile membranous structure, called the basilar membrane, which divides the cross section of the cochlea almost in the middle. The basilar membrane plays a crucial role in the biomechanics and hydrodynamics of the inner ear and, therefore, in the mechanism of sound sensation. Located on top of the basilar membrane is the organ of Corti, which includes the hair cells and forms the neural receptor for sound waves. Physical integrity of the basilar membrane is thus essential for acoustic hearing.

**Figure 1:**
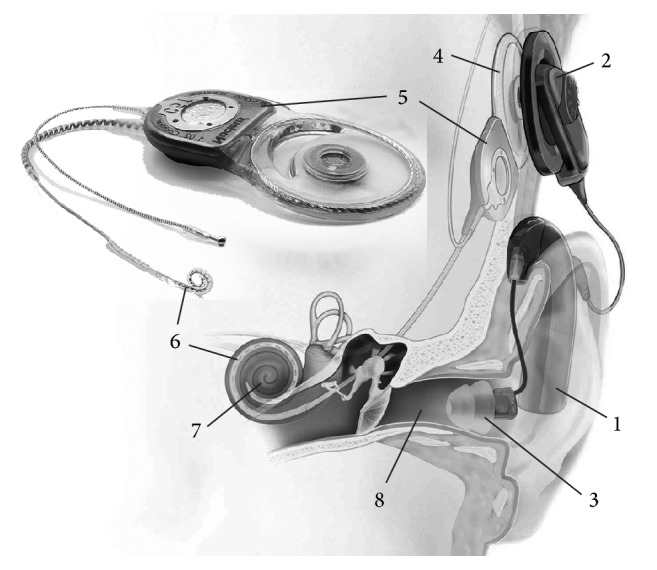
Cochlear implant system for hybrid stimulation. It consists of external (1–3) and internal (implanted) components (4–6). Sounds are captured and digitized by the external sound processor (1, including one or more microphones). Signals and energy are transcutaneously transferred to the implanted portions using an external (2) and an internal (4) coil. These signals are converted by the implant (5) into stimulus-correlated electrical pulses and transmitted to the electrode array (6) implanted into the cochlea (7). In this way, electric stimulation evokes neural responses in the intact auditory nerve. Additionally, low-frequency sound is amplified by the sound processor (1) and transmitted to the normal hearing pathway using an earmould (3) in the external auditory canal (8) (images by courtesy of Cochlear Ltd.).

In hearing-impaired patients, the function of the hair cells, which convert acoustic signals into a neural response, is limited or completely absent. The latter case results in deafness. Otherwise, the degree of hearing loss depends on the amount of residual hearing and its usability for communication and environmental sound sensation. However, a useful and well-established surgical procedure—the implantation of an electronic device called a cochlear implant (CI, see Figure 1)—is available to treat deafness and profound to severe hearing loss.

Through electrical stimulation of the auditory nerve, a CI bypasses damaged portions of the ear and provides a sense of sound to the recipient. For this purpose, the CI system includes a component known as the electrode array (also referred to as the electrode carrier or simply the electrode), which is inserted into the cochlea (the terms “cochlea,” “inner ear,” and “scala tympani” are subsequently used synonymously; in all cases, what is meant is that the electrode array should be ideally inserted into the scala tympani, irrespective of the actually achieved outcome). This intracochlear portion is a thin, elongated silicone body incorporating embedded platinum contact electrodes. To achieve electrical stimulation of the auditory nerve, environmental sounds are recorded and converted into electrical signals by the CI system. Stimulus-correlated electrical signals are finally transferred to the electrode array; this in turn generates electrical fields in the surrounding tissue, including the fibres of the auditory nerve.

Historically, only deaf patients were considered to be candidates for CI treatment. In the present day, inclusion criteria are continuously being expanded, so that individuals with substantial residual hearing are now also getting implants. There are two main reasons why a CI is recommended to these patients. The first is that, in most cases, residual hearing is in the low-frequency range. In contrast, hearing at the high-frequency range (>1,000 Hz), including those frequencies essential for human speech, is not functional. These individuals are, therefore, excluded from day-to-day communication, a situation which cannot be improved by use of conventional hearing aids. The second reason is a strategy referred to as hybrid or electric acoustic stimulation (EAS). This involves the use of electrical stimulation via the CI to restore high-frequency hearing, with the residual hearing still being exploited and amplified for normal (acoustic) low-frequency hearing. A combination of both approaches to providing hearing sensation is known to deliver better hearing outcomes than electrical stimulation alone [1–7]. This is why EAS is currently one of the most important objectives in CI treatment and an important motivation for research. This leads us back to anatomical aspects of CI surgery, as the integrity of the basilar membrane after electrode insertion is essential for residual hearing preservation and thus for electric acoustic stimulation.

In general, existing electrode arrays can be divided into two main groups: those straight in shape and those with a preformed, moulded silicone body. Numerous straight electrode arrays have been developed in recent years. High flexibility, reduced cross-sectional area, and limited insertion depth are considered as essential features to meet the requirements of atraumatic insertion, and hence preservation of residual hearing and electric acoustic simulation. The main disadvantage is the final position at the lateral wall of the cochlea, which is a large distance away from the neural tissue that is the target for stimulation.

In contrast, preformed electrode arrays are fabricated in a spiral configuration adjusted to the shape and size of an average human cochlea. As insertion into the cochlea initially requires a straight configuration, it is necessary to uncurl the electrode array and keep it straight prior to insertion. A commonly used mechanism is a thin but sufficiently stiff wire (stylet) inside the electrode array, which inhibits the curling forces of the spirally preformed and elastically deformed silicone body and the embedded platinum wires. During insertion this stiffening wire, the stylet is removed and the electrode array returns to its original helical shape.

Originally, these preformed electrode arrays were designed for intracochlear placement next to the modiolus, which is why they are known as perimodiolar implants. The modiolus is the central axis of the cochlea and contains the neural tissue that is the target for stimulation by the CI. Therefore, close proximity of the electrode contacts to the inner wall of the cochlea is beneficial for electric stimulation [8, 9]. Examples of this type of perimodiolar implant are the Contour Electrode, its successor the Contour Advance electrode, and the thinner Modiolus Research Array (MRA), all three of which are manufactured by Cochlear Ltd. (Sydney, Australia), and the HiFocus Helix electrode made by Advanced Bionics LLC (Valencia, CA, USA).

However, this perimodiolar design also has its drawbacks. In general, these electrode arrays have a larger diameter and (due to the stiffening wire) also exhibit greater overall stiffness than the thin and highly flexible straight models. Perimodiolar electrode arrays are thus associated with a higher risk of insertion trauma and are not normally used for patients with residual hearing. To overcome the disadvantages of additional stiffening elements (such as the stylet) with regard to residual hearing preservation, it would appear necessary to reduce the amount of contact between the implant and the intracochlear anatomical structures. One strategy could be to use robot assistance for insertion and to optimize its pose (position and orientation) with respect to the pose of the inner ear [10, 11]. Reduced contact implies reduced insertion forces, which are commonly accepted as being correlated to insertion trauma.

**Figure 2:**
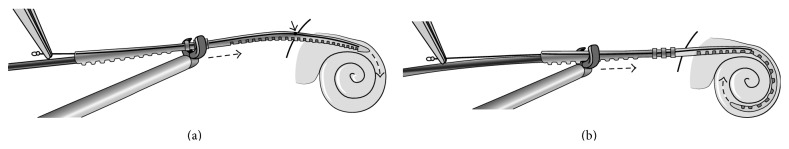
Schematic illustration of the Advanced Off-Stylet (AOS) technique. (a) The electrode array is inserted into the inner ear with the stylet inside until a marker is at the level of the cochleostomy site. (b) The stylet is kept stationary and the implant is advanced further into the cochlea until full insertion depth is achieved (image provided by courtesy of Karl STORZ, Tuttlingen, Germany).

However, the integrated curling mechanism provides the opportunity to modify not only the pose but also the shape of the electrode array with regard to the individual anatomy. This entails the replacement of a uniform insertion technique (such as the Advanced Off-Stylet (AOS) technique; see Section 2.2.2 and Figure 2) by a patient-specific one. This is, of course, possible only on a limited scale as the commercially available perimodiolar implants are not developed and designed for this purpose, and additional intraoperative surgical assistance devices are necessary because an individually optimized insertion process can no longer be performed manually. Nevertheless, thanks to recent advances in surgical master-slave systems [12], and especially in robot-assisted devices for CI surgery [13–18], as well as in automated insertion tools [11, 19–25], it seems to be only a question of time until accurate assistance devices can be used intraoperatively for electrode insertion.

Therefore, the aim of this study was to investigate whether, for the commercially available CA electrode array, an individually optimized insertion procedure is advantageous compared with the AOS insertion technique, which is recommended for all patients by the manufacturer, Cochlear Ltd. More generally, we wished to explore whether taking into account the curling behaviour of a CI electrode array is beneficial in terms of less traumatic insertion. In the light of this research question, we also investigated the situation when a minimally invasive approach to the cochlea is employed. This entails an alternative to the manually performed drilling of a large mastoid cavity (a procedure known as mastoidectomy, which involves the removal of parts of the temporal bone behind the ear to provide access to the inner ear). Instead, a single, straight drill hole should be used. This not only requires additional surgical assistance devices, which are currently under development by several research groups [13–15, 17, 18, 26–28]: a hole of such small size also means certain constraints in terms of electrode insertion, especially loss of several degrees of freedom.

Irrespective of the surgical approach chosen, the findings of this research on individually optimized electrode insertion are also of interest for other projects dealing with the functionalization of CI electrode arrays using different curling mechanisms. In recent years, there has been an increasing interest in “active” or “steerable” CI electrode arrays, which involves investigation of different actuator principles to bring about a controllable shape change. The following are examples of these mechanisms: use of an embedded actuation strand as described by Zhang et al. [24] and Zhang and Simaan [25], shape change of the silicone body by varying the pressure of an internal fluid as independently described by Arcand et al. [29] and Zentner [30], and the use of “smart materials” such as thermal shape-memory alloys. In the latter, nickel-titanium (NiTi) alloys play a dominant role. One option is to use a single NiTi wire inside the electrode array, covering almost its entire length to provide a desired final shape [31, 32]. Alternatively, the integration of multiple, separately activatable actuators (in terms of an actuator array) is described [33, 34], the aim of which is to bring about a spatially resolved change in implant shape as, for example, via electrical resistance heating.

## 2. Materials and Methods

### 2.1. Hypothesis and General Approach

In contrast to manually controlled insertion, the automated insertion tool provides a means of adjusting the electrode's curling behaviour to the individual helical shape of the cochlea. The following working hypothesis was therefore proposed:

*Individual adjustment of the curling behaviour of a preformed CI electrode array to the individual spiral shape of the cochlea can improve the insertion process in terms of reduced risk of insertion trauma.*

In order to test the hypothesis, a principle subject to general consensus in CI research was applied, according to which insertion forces are the main indicator of the risk of insertion trauma and are directly correlated to it. From a mechanical point of view, however, insertion forces are primarily a result of contact or constraining forces between the implant and the surrounding anatomy. Thus, the degree of contact between both “objects” allows a qualitative conclusion concerning the insertion forces and ultimately, therefore, concerning the risk of trauma. As a first step towards the substantiation of the working hypothesis, proof has to be provided that, by means of the controlled and continuous adjustment of the shape of the electrode array to that of the cochlea, the degree and severity of contact between them can be reduced. This enables a direct conclusion to be made concerning reduction of trauma risk.

For this purpose, the geometry and the curling behaviour of a commercially available CI electrode array were modelled, based on experimental data from a previous study [10]. Additionally, the anatomy of a representative number of human cochleae was modelled and brought together in a custom-made simulation tool called SimCInsert. Using this tool, the insertion process was simulated for the conventional manual procedure (both standard and minimally invasive access) and compared with full, manually optimized insertion. The risk of trauma was evaluated in all cases and discussed in the light of the working hypothesis.

### 2.2. Modelling the Preformed Electrode Array

#### 2.2.1. Contour Advance Electrode Array

The Contour Advance (CA) is a well-known and widely used preformed electrode array. Its silicone body is moulded in a precurved shape. Twenty-two half-banded platinum electrode contacts are embedded in it, each connected via a 25 *μ*m thin platinum wire [35]. Therefore, the stiffness of the electrode array increases from the tip to the basal portion as the number of wires enclosed in the silicone increases. This stiffness gradient is reinforced by the decreasing diameter of the electrode array, which tapers from 0.8 mm down to 0.5 mm at the tip. The tip of the implant has a conical shape and was designed to reduce contact forces during insertion. This special feature of the CA is known as the Softip. A white marker between electrodes 10 and 11 (7.6 mm behind the tip) is a visual aid for the surgeon for estimating the insertion depth and was integrated to assist the execution of the Advance Off-Stylet (AOS) insertion technique. Finally, three silicone ribs at the end of the intracochlear portion indicate full insertion depth and facilitate sealing of the cochlea after insertion due to soft-tissue growth.

All electrode arrays employed in this study were provided by the manufacturer Cochlear Ltd. (Sydney, NSW, Australia). They were rejected during the production process owing to electrical defects. With respect to mechanical properties, which are of relevance for this study, these electrode arrays are, however, identical to commercially delivered and clinically used ones. They differ from the (also available) practice electrodes also provided by Cochlear Ltd. as, for example, part of surgical practice kits for teaching purposes and training courses. These practice electrodes are produced by a simpler manufacturing technique to be less expensive but therefore exhibit different curling behaviour than that of “real” implants. Practice electrodes were thus not usable for this kind of investigation [10].

#### 2.2.2. AOS Technique

Together with the new electrode array, the AOS insertion technique was introduced to standardize the manual insertion procedure and to provide the surgeon with a straightforward and reliable modus operandi. This launch of a “self-curling” electrode array [35], in conjunction with a special insertion technique, was therefore considered an important and valuable step conducive to decreasing the risk of soft-tissue trauma due to insertion. The AOS insertion technique involves keeping the CA electrode array straight and inserting it into the cochlea with the stylet inside until the white marker reaches the opening of the inner ear (cochleostomy). It is assumed that the white marker indicates that the tip of the implant reaches a central position within the basal turn of the cochlea. As a second step, the actual AOS phase, the stylet is kept stationary by the use of a small pair of tweezers and the electrode is advanced off it. Owing to internal bending stresses, the electrode array returns to its original shape and thus curls around the inner wall of the cochlea (modiolus).

#### 2.2.3. Determination of Curling Behaviour

**Figure 3:**
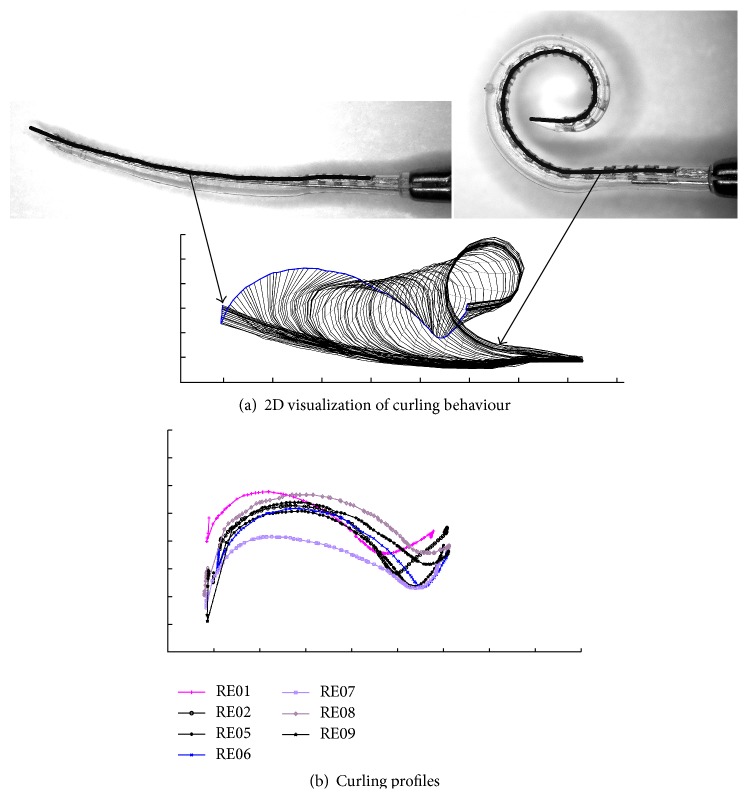
(a) Visualization of the curling behaviour of a preformed Contour Advance electrode array (RE06) using the 22 detected platinum contacts and the location of the Softip. The start configuration (on the left side) with stylet inside is characterized by a nearly straight configuration (compared with Figure 2(a)). Due to stylet extraction, the electrode array returns into its preformed spiral shape (right). By tracking the complete range of curling behaviour, the movement of the tip of the implant shows a typically sigmoidal curve. This curve is indicated using a bold blue line and is referred to as the curling profile of the electrode array (here RE06). Scale marks indicate 1 mm. (b) After determination of all curling profiles, four electrode arrays were selected and used in this study, which together cover the full range of curling behaviour investigated. RE01 and RE08 represent electrode arrays with a highly pronounced curling behaviour, measured as deflection of the tip from the straight configuration. RE06 was chosen to represent a moderate curling profile and RE07 to represent the flattest one.

The curling behaviour of several CA electrode arrays has already been determined in a former study [10]. Using a custom-made micromanipulator, the stylet was extracted in increments of 0.1 mm to 0.25 mm. After each step, the resulting shape of the CA electrode array was digitally documented using a reflected-light microscope (MZ 6, Leica Microsystems GmbH, Wetzlar, Germany, in conjunction with DS-L1, Nikon Cooperation, Tokyo, Japan). In this way, a series of images was generated which records the curling behaviour of each implant. For further processing of these images, a semiautomatic image-processing procedure was developed and applied to identify the centre of all 22 platinum contacts as well as the tip of the silicone body. The second step involved fitting a mathematical function, consisting of a logarithmic spiral and up to three straight segments, through the points. Hence, the actual shape of the electrode array (depending on the extent to which the stylet is removed) was finally modelled by a continuous curve.  Figure 3 shows the visualization of one CA electrode array as a result of stylet extraction. After projection in the *x*-*y* plane, the movement of the electrode tip exhibits a typical sigmoidal curve (highlighted in blue in Figure 3(a)). This “curling profile” of each electrode array was used to compare the differences in the specific curling behaviour of different arrays.

To allow a representative investigation, four electrode arrays were selectively chosen from all measured implants referred to in [10], in order to cover the total range of known variability in curling behaviour. After determination of all curling profiles (the rigid gripping of the electrode array serving as the common reference), RE01 and RE08 were selected as examples of highly pronounced curling behaviour, measured as deflection of the tip from the straight configuration (see Figure 3(b)). RE06 was also chosen to represent a moderate curling profile and RE07 to represent the flattest curling profile.

#### 2.2.4. Modelling the Electrode Array

**Figure 4:**
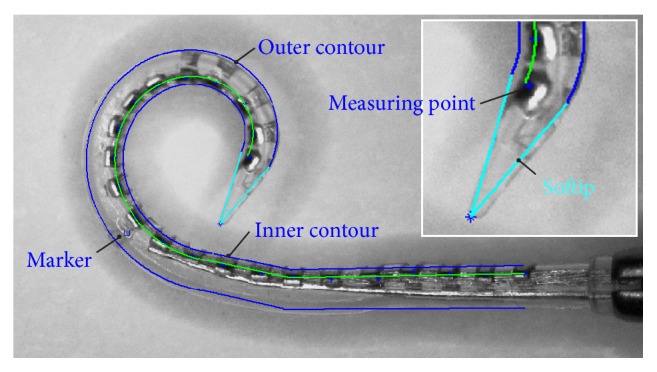
Overlay of an original image of the electrode array and its modelled shape.

Based on these already existing data, the spatial dimensions of the electrode array in the curling plane were modelled. This was necessary for meaningful simulation of the intracochlear curling behaviour since, if the shape of the electrode array is represented only by a thin line, it is not sufficient to allow the interaction of the implant with the surrounding anatomical structures to be modelled. Therefore, the inner contour of the CA electrode array was modelled by drawing a second polyline at a constant distance of 0.15 mm to the fitted central path. The same applied to the outer contour of the electrode array, with decreasing distance between 0.65 mm and 0.45 mm to allow for the tapered shape of the silicone body. The tip was added to the outline by means of two straight lines. This approach to visualizing the changing geometry of the electrode array caused by stylet removal was verified by overlaying the original images with the drawn outline of the implant. Good agreement between the calculated shape of the electrode array and the experimental images was obtained, as shown in Figure 4.

### 2.3. Modelling the Inner Ear

#### 2.3.1. Imaging and Segmentation

Image data and segmentations of 23 human temporal bone specimens were available from former studies. These data were acquired using flat-panel-based volume computed tomography (fpVCT, GE Global Research Center, Niskayuna, NY, USA) [37, 38] at the Department of Diagnostic Radiology, Goettingen University Hospital (Goettingen, Germany). This experimental device allows higher resolution (approximately 200 *μ*m, isometric) than does customary, clinically available computed tomography (CT) scanners. Using a threshold-based segmentation algorithm and manual refinement (iPlan 2.6 ENT, BrainLAB AG, Feldkirchen, Germany), 3D models of the human inner ear had been generated. Segmentation covered the entire bone-embedded spiral canal of the cochlea and not merely the scala tympani, as the basilar membrane is not visible in X-ray-based imaging. Each 3D model of the inner ear had been saved as STL files (Standard Tessellation Language, a popular data format for 3D surface models).

**Figure 5:**
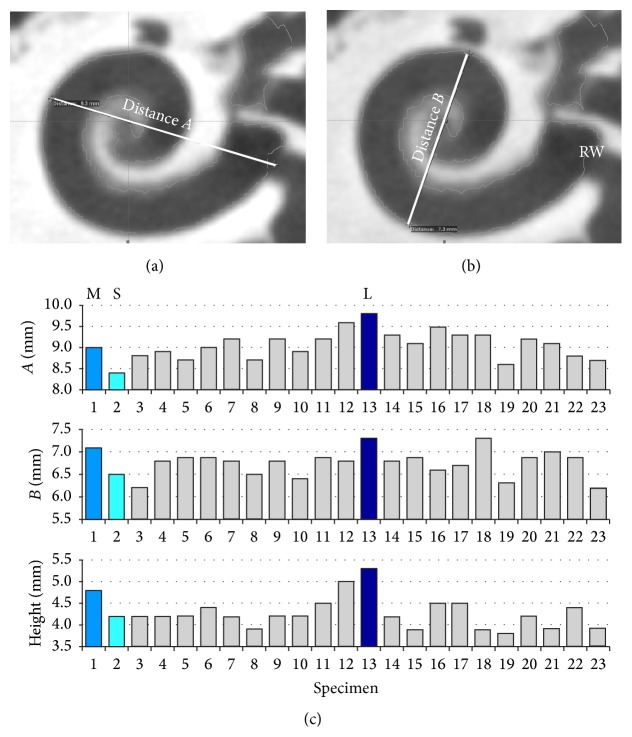
(a, b) Example images showing the measured distances *A* and *B* according to the metrological method introduced by Escudé et al. [36]. RW: round window membrane. (c) Bar chart showing *A* and *B* values and height of the inner ear for all 23 investigated cochleae. Distance *A* was used to distinguish the smallest, largest, and medium-sized cochlea.

The present study is designed to take into consideration the anatomical variability of the human inner ear. In order to have a single parameter which characterizes differences in size, a measurement method introduced by Escudé et al. [36] was applied. The greatest lateral dimension of the basal turn was measured (distance “A”) in accordance with their description (see Figure 5). Based on these values, the smallest (CS), the medium-sized (CM), and the largest (CL) cochleae were chosen and used for the subsequent investigations as representatives of inherent anatomical variability.

#### 2.3.2. Transformation into 2D

The CA electrode investigated shows planar (two-dimensional, 2D) curling behaviour. This is common in perimodiolar cochlear implants for reasons of cost-effectiveness, as 3D curling behaviour requires separate products for left and right ears. The modelling of insertion behaviour was, therefore, initially also considered as a 2D problem. This leads to the necessity of transferring the 3D volume data for the three selected cochleae into a 2D representation of their geometry. For this purpose, custom-made software was employed (courtesy of Mr. A. Hussong, Institute of Mechatronic Systems, Leibniz University of Hannover), which was developed using C++ in Visual Studio (Microsoft Corporation, Redmond, WA, USA) and VTK (Kitware Inc., Clifton Park, NY, USA). This program enabled the following steps:

1. A segmented cochlea was loaded as an STL file.
2. A rotation axis through the modiolus was manually determined. It was employed to calculate a cutting plane which provides a cross-sectional view of the cochlea.
3. This cutting plane can be rotated in equal steps (here, 5° steps were used), starting with a plane which passes through the round window niche.
4. Two points were manually selected for each resulting cross-sectional view. One marked the outer and the other the inner contour of the cochlea.

**Figure 6:**
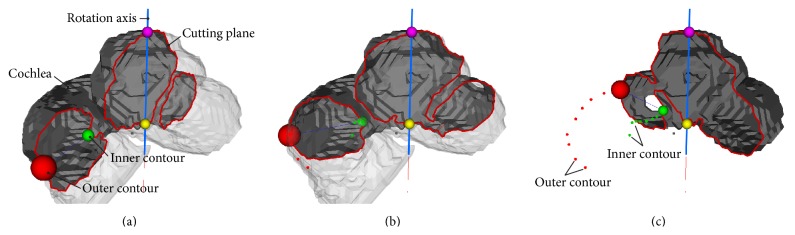
Principal procedure involved in transforming the three-dimensional geometric model of the cochlea into a two-dimensional one. A cutting plane, rotated around the central axis (modiolus), provides stepwise visualization of the cross sections. In each cross section, a point on the outer contour (red) and a second one on the inner contour (green) were manually marked. After perpendicular projection onto a common plane, the points in their totality described the geometry of the inner ear in a 2D manner.

By repeating steps (3) and (4), the spatial dimensions of the helical cochlear lumen were recorded as a 3D point cloud. Finally, these points were projected onto a plane (an orthogonal-distance regression plane) in order to obtain 2D curves describing the inner and outer contours of the cochlea (see Figure 6).

### 2.4. Modelling the Insertion Process

#### 2.4.1. SimCInsert

**Figure 7:**
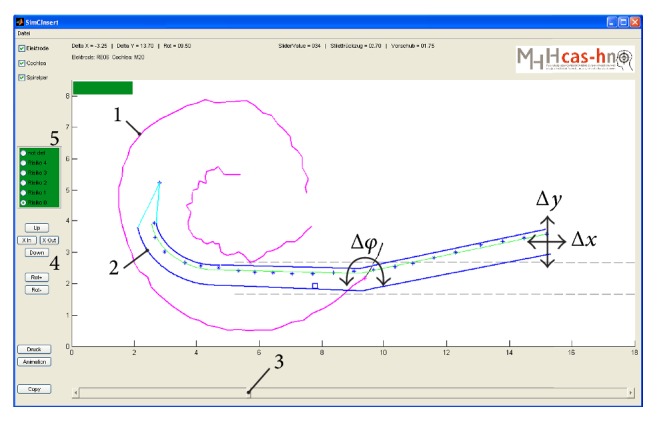
Graphical user interface (GUI) of the custom-made simulation tool “SimCInsert.” It enables visualisation of both the geometrical data for the investigated inner ears (1, here: medium-sized) and the shape of the electrode arrays (2, here: RE06). Using the slider (3), the stylet (not visualized) can be virtually moved; that is, the array's shape is manipulated. Using six cursor buttons (4, two for each interactively adjustable parameter Δ*x*, Δ*y*, and Δ*φ*), the location and orientation of the electrode array for each shape can be manipulated with respect to the cochlea plotted at the same (fixed) position. Use of both input options enables a complete insertion process to be simulated. Radio buttons (5) were included to interactively rate the risk of insertion trauma (see Section 2.5).

Both in order to model intracochlear curling behaviour and for the intended optimization of the insertion process, a simulation tool called “SimCInsert” was developed using MATLAB (R2008b, MathWorks, Natick, MA, USA). The corresponding graphical user interface (GUI) is shown in Figure 7. The GUI allows loading of the prepared cochlear contours (see Section 2.3.2) which are visualized at a constant (fixed) position within the main window. In contrast, the visualization of the electrode array (also loaded via a task menu entry) is dynamic and based on the stored data on curling behaviour as a function both of stylet extraction and of interactively adjustable parameters for the position (Δ*x*, Δ*y*) and orientation (Δ*φ*) relative to the cochlear contour. By using a slider at the bottom of the GUI, the user is able to control stylet retraction. The corresponding configuration of the electrode array is automatically loaded from the database (see Section 2.2.3). These “raw data” are transformed according to the manually chosen location parameters. As choosing the desired position and orientation of the electrode array relative to the cochlea's geometry involves a 2D task, there are displacements in both *x* and *y* directions, as well as one rotation around the cochleostomy. These “transformed data” are extended to the 2D representation of the electrode array as described in Section 2.2.4 and visualized in addition to the cochlear contour within the main window. Via translation in the negative *x* direction, for example, the feeding of the implant into the inner ear is simulated in SimCInsert (which is equal to insertion depth). In this way, SimCInsert provides a fairly simple means of simulating the conventional AOS technique, while also allowing manual optimization of the insertion process.

#### 2.4.2. Simulation of the Manual AOS Technique (manAOS)

The conventional, manually performed AOS insertion technique, as recommended by the CI manufacturer Cochlear Ltd., served as a reference for the subsequent optimization of the insertion process. In this case, only the second phase, with actual stylet extraction, was taken into account. However, the instructions for the AOS insertion technique are idealized. In reality, the CA electrode array shows a banana-shaped starting configuration, as opposed to a straight one, even where the stylet is fully plugged in. Therefore, a completely rectilinear/linear insertion into the basal turn (without contact with the cochlear walls) is in reality not possible. Furthermore, each implant initially has a slightly different shape [10].

Thus, for the simulation of the manually performed AOS technique, it was assumed that the surgeon adjusts the insertion process (irrespective of whether this is done intuitively or in a controlled manner). Although there are no “hard data,” this assumption is confirmed by observations in the operating theatre during cochlear implant (CI) surgery and the verbal reports of CI surgeons indicating that there is a slight compensatory movement when the initially curved electrode array is passed through the cochleostomy or the round window access. This manual adjustment is made possible by use of visual information through the surgical microscope, as well as haptic feedback. During the simulation, this compensatory movement was represented by a rotation in the *xy* plane. In this paper, the simulation of the manually performed AOS insertion technique is subsequently referred to as “manAOS.”

#### 2.4.3. Simulation of the Automated Approach (autoAOS)

The situation modelled as a second scenario involved accessing the inner ear by means of automated insertion through a minimally invasive drill canal. In this case, the electrode array is gripped behind the last platinum contact and is advanced in a strongly linear fashion into the cochlea inside the guiding tube of the insertion tool. In terms of the modelling in SimCInsert, this means that throughout the remainder of the insertion process, values for both the position in the *y* direction (Δ*y*) and the rotation (Δ*φ*) of the electrode array had to be kept constant after initial positioning. Only movement in the *x* direction (Δ*x*) was used to insert the electrode array into the cochlear contour to the same extent as the stylet extraction. For evaluation of minimally invasive access, two parallel grey dashed lines in the main window of SimCInsert represent the edge of the drill hole or the guiding tube of the insertion tool.

#### 2.4.4. Optimization of the Insertion Process (optIns)

Finally, the insertion of the CA electrode array was manually optimized and tailored to the three different sizes of cochleae, representing the anatomical variation in human individuals. All insertion parameters could, therefore, be modified in order to optimize the location of the electrode array within the cochlea. In particular, it was no longer necessary to maintain the strong linear relationship between stylet extraction and implant feed as specified by the AOS technique.

According to the initial working hypothesis, the aim of the optimization was to optimally tailor the curling of the implant to the individual anatomy of the inner ear at each stage of the overall insertion process. An optimum location within the cochlea was indicated by minimal overlap between the contour of the electrode array and the contours of the inner ear. A small amount of overlap was considered as equivalent to low contact forces (and hence low forces pertaining to insertion trauma) and vice versa.

This manual optimization was repeated for each stored configuration of the electrode array, that is, for all steps of stylet removal. Additional constraints were monotonic increase of implant feed (which means Δ*x*), full insertion with complete stylet removal, and avoidance of abrupt changes in all insertion parameters. The latter was to ensure a well-adjusted and continuous insertion process which could be implemented by using, for example, an automated insertion tool and its programmable linear drives [19, 39]. Subsequently in this paper, the abbreviation “optIns” is used to refer to optimized insertion.

### 2.5. Trauma Risk: A Rating Scale for Risk of Insertion Trauma

To allow comparative evaluation of these three different scenarios, especially comparison between individually optimized insertion and the conventional approach, a useful assessment procedure needed to be developed. Although it is known that “objective” measurement methods are generally considered to be more reliable than “subjective” ones, in this special case, a subjective rating scale was (on the strength of various arguments) introduced and preferred (discussed in detail in Section 4.3). The main argument is that there is a lack of computational structural mechanics. This means that deformation of the electrode array due to direct contact with the boundary of the inner ear cannot be simulated and correctly visualized. The main advantage of the new rating scale designated “trauma risk,” explained below, is that the user is able, to a certain extent, to compensate for this lack of deformation simulation by their mechanical expertise. This allows holistic assessment of the insertion-related risk of trauma to the inner ear.

After Eshraghi et al. [40], a rating scale was introduced, known as “trauma risk,” which extends between the grades 0 (no contact between implant and inner ear) and IV (extensive contour damage). Details can be found in Table 1. The classification was chosen based on the assumption that trauma risk of grades 0 and I has no negative influence on residual hearing preservation. Beginning with grade II, the risk of iatrogenic hearing loss or deafness increases, which is very likely at grade IV.

**Figure 8:**
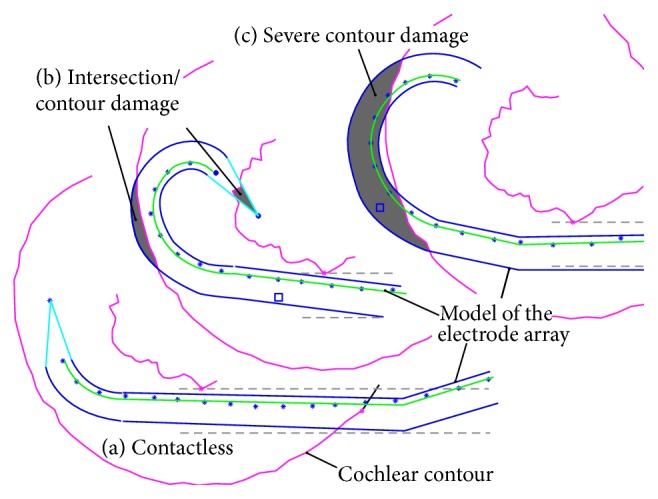
Examples of different kinds of contour damage/intersection. (a) Without contact between electrode array and cochlear contour (grade 0). (b) Trauma risk grade II with both penetration of the electrode tip into the inner wall and overlapping of the silicone body with the outer wall. In fact, both lead to relevant contact forces inside the inner ear. (c) Extensive contour damage which results in the highest risk of causing an insertion trauma (grade IV).

**Figure 9:**
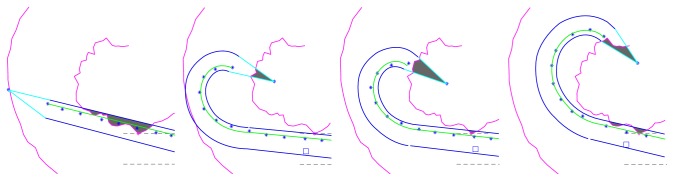
Examples of trauma risk grade II.

Using this risk-rating method for each step of the modelled insertions, the degree of conformity of the implants' shape and the shape of the inner ear was rated (see Figures 8 and 9). Ideally, the electrode array lies fully inside the contours of the cochlea without any intersection. This means contact-free insertion with no insertion forces and, therefore, no insertion trauma. Mechanical contact between the implant and the inner ear (resulting in contact forces and thus a corresponding risk of insertion trauma) is visualized as an intersection (damage) of the contour of the electrode array and the cochlear contour. The extent of contour damage allows a qualitative valuation of the corresponding risk of intracochlear trauma and therefore loss of residual hearing. However, the “trauma risk” rating scale introduced is not, unlike Eshraghi et al.'s [40] “trauma grade,” a criterion for postexperimental evaluation. Rather, it is a method of estimating the risk of insertion trauma in advance (i.e., prospectively). Limitations of this evaluation method are discussed in detail in Section 4.3.

## 3. Results

**Figure 10:**
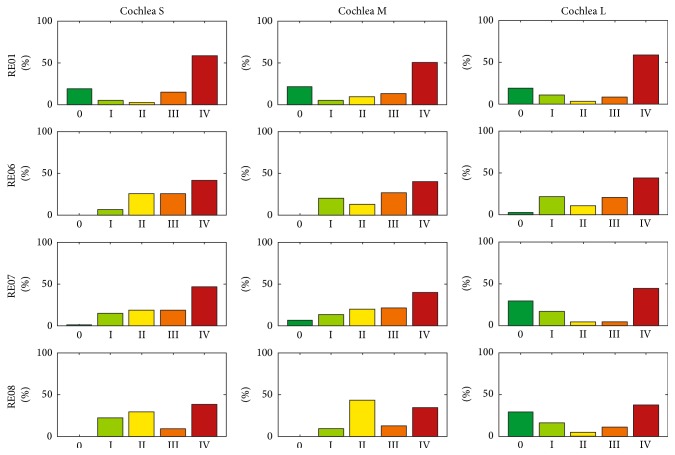
Histogram of the distribution (observation frequency) of the different trauma grades for each modelled insertion, where the procedure is performed manually (manAOS). To improve comparability, all results are normalized by reference to the total numbers of investigated steps in the insertion process.

**Figure 11:**
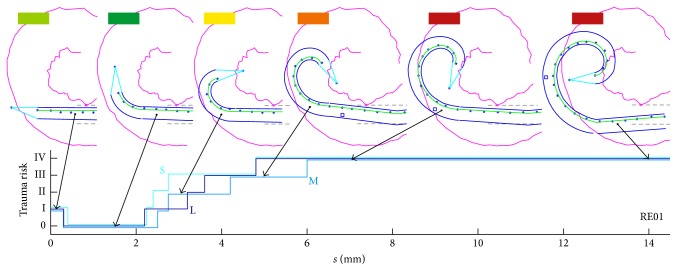
Change of trauma risk during the manually performed insertion process (manAOS), plotted for RE01 as an example, with corresponding screenshots from SimCInsert. Trauma risk is plotted against stylet extraction *s*.

In total, 36 insertions were modelled using three different cochleae (small, medium, and large) and four different electrode arrays in order to compare three different scenarios. As a reference, the manual procedure for inserting the Contour Advance (CA) electrode array using the Advance Off-Stylet technique (manAOS) was remodelled using SimCInsert. Applying the above-mentioned assumptions about the intuitive adjustment of the insertion process, the trauma risk of the insertion process is distributed as shown in Figure 10. Irrespective of the specific electrode array and the size of the cochlea, a high proportion of the total process is characterized by a risk of trauma of grade ≥ II. More detailed information, using RE01 as an example, is provided in Figure 11. Differentiated according to the three different-sized cochleae, the trauma risk is plotted against stylet extraction (which is inversely proportional to insertion depth). Sample images from SimCInsert show the corresponding insertion depth as well as the contour damage. This schematic illustration demonstrates that insertion is initially less traumatic (in the basal turn of the cochlea) than with progressive insertion depth.

**Figure 12:**
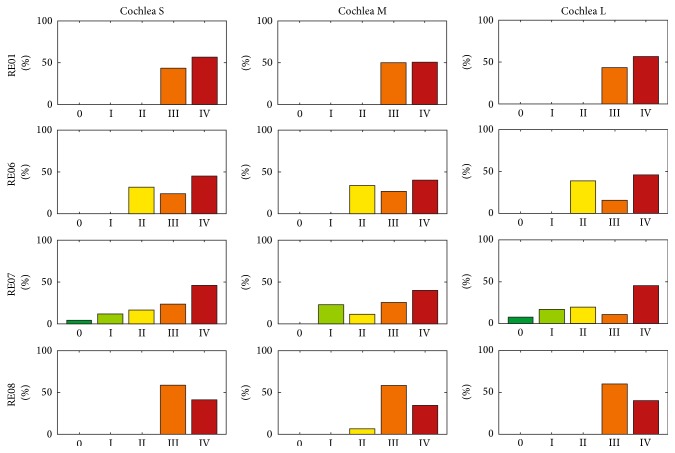
Histogram of the distribution (observation frequency) of the trauma risk where the procedure is performed in an automated manner. As no additional parameters could be adjusted to tailor the orientation of the electrode array, the simulation of the automated AOS technique (autoAOS) represents its most consistent implementation in this study. The histogram clearly shows the high portion of trauma risks III and IV on the insertion process. Only with RE07 is less trauma risk (≤I) observed. Of the electrode arrays in the study, RE07 is the one with the flattest curling curve and pronounced straightening in the initial phase.

In contrast to the manual approach, the automated procedure is inadequate with regard to the necessary adjustable positioning and orientation, especially when using minimally invasive access to the inner ear. Simulation of the autoAOS scenario results in the trauma risk as shown in Figure 12. This figure clearly illustrates that a high proportion of phases are rated as high-risk (i.e., trauma risk ≥ III). For RE01 and RE08, the insertions were rated as potentially harmful in almost all cases. The RE07 electrode array is the exception; at least in some phases of the insertion process, the actual shape of the implant fits the anatomy of the cochlea, resulting in no or only minimal intersection of both contours.

**Figure 13:**
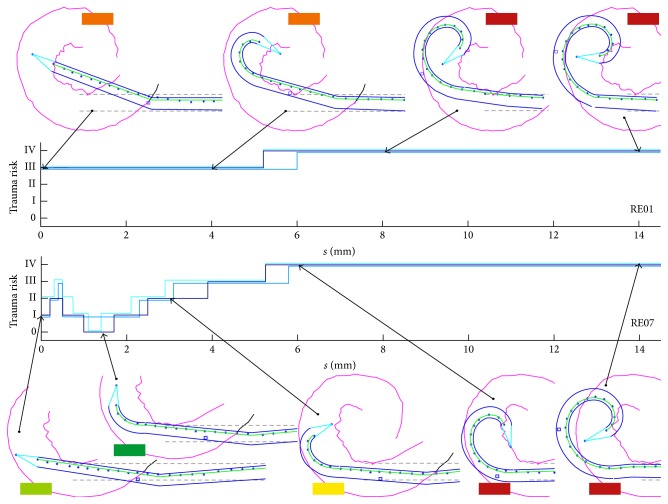
More detailed findings with regard to trauma risk throughout the insertion process for RE07 compared with RE01. The results indicate that where no additional adjustment of the electrode orientation is possible, a straight starting configuration is more advantageous than the slightly curled one.

Figure 13 provides detailed insight into the changing rating of the trauma risk with progressive insertion depth. For this purpose, the two extreme cases (RE01 and RE07) are plotted together in one figure to show the overall spectrum of the results and its correlation with the curling profile of the electrode array. While the insertion of RE01 is completely in the orange or red zone, the trauma risk using RE07 is at least reduced in the initial phase of insertion. It is striking that RE01 shows very pronounced deflection of the electrode tip from the ideal straight configuration, which means a marked initial curvature (starting configuration). In contrast, the profile for the curling behaviour of RE07 is very flat (see Figure 3). Based on this finding, it is concluded that the good straight starting curvature is causal and thus beneficial in terms of low trauma risk during the initial phase of the insertion process.

**Figure 14:**
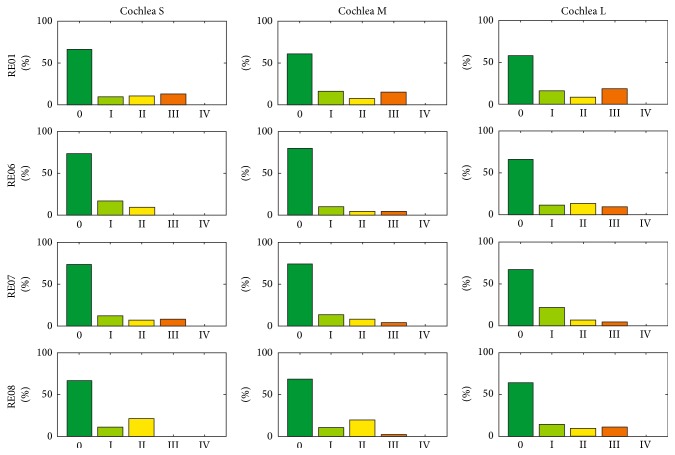
Histogram of the distribution (observation frequency) of the trauma risk after individual optimization of the complete insertion process (the AOS part). For all examined implants and inner ear geometries, it was possible to markedly reduce the risk of insertion trauma. Compare this figure with (optIns) Figure 10 (manAOS) and Figure 12 (autoAOS). The poorest outcomes are observed with RE01. However, after adjusting the curling of the electrode array to the surrounding anatomy, the insertion process is subject to trauma risk of grade 0 for all investigated cases.

While insertion processes using the conventional AOS technique are mainly characterized by trauma risk grades III and IV, it is possible to reduce the risk of insertion trauma by means of the individual optimization strategy introduced.  Figure 14 shows the percentage distribution of the trauma risk assessment for the optimized insertion (optIns). In comparison with manAOS (see Figure 10) and minAOS (see Figure 12), it is evident that the overall insertion process is shifted toward less traumatic implantation. After optimization, by tailoring the specific curling behaviour of the electrode array to the individual anatomical constraints, the insertion process is chiefly characterized by trauma risk grade 0. In all 12 investigated cases (combinations of differing curling behaviour and cochlear anatomy), the rating of the trauma risk with grade 0 has the largest share.

**Figure 15:**
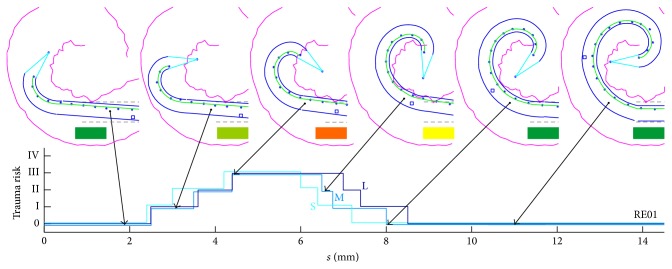
More detailed findings with regard to change in trauma risk during an optimized insertion. RE01 was chosen because, of all the optimizations, it represents the “worst case”.

The intracochlear curling caused by the stylet extraction and resulting from the individual optimization process is shown in Figure 15, with RE01 used as an example. As RE01 is the electrode array with the poorest trauma risk rating for all optimized insertions, Figure 15 shows the “worst case.”

## 4. Discussion

Atraumatic insertion is a mandatory prerequisite for hybrid stimulation of patients with residual hearing. Generally, reducing the risk of iatrogenic trauma requires that the amount of interaction and resulting contact forces between the implant (here the intracochlear electrode array) and the surrounding anatomical structures (here especially the basilar membrane and other delicate soft-tissue structures) be diminished. One established strategy in cochlear implantation is to design very thin and flexible electrode arrays. This is done to limit the contact forces resulting from the extensive contact between these straight implants and the lateral wall of the cochlea. Another design strategy takes the shape of the inner ear into account: by producing helically preshaped electrode arrays, the aim from the outset was to reduce the amount of contact with the boundaries of the scala tympani. This was the purpose and motivation behind the introduction of the Contour Advance (CA) electrode.

The new approach to reducing the risk of insertion trauma taken in this study goes beyond the isolated modification of the design of the electrode array. Instead, the essential aspects are, firstly, the individualization of the insertion process, which entails the consideration of individual anatomical constraints and, secondly, the optimization of the insertion process by tailoring the change in shape of the implant to the shape of the surrounding hollow organ.

### 4.1. Comparison with the Literature

The necessary investigation of the CA's curling behaviour has already been performed and published [10]. In the meantime, comparable investigations have been conducted by Pile et al. [11], who also used stepwise extraction of the stylet from a fixed electrode array. Each step, with 1.27 mm increments (those in the present study being 0.1 mm to 0.25 mm), was digitally captured; the location both of the platinum contacts and of the electrode tip was manually marked and served as reference points for a curved line, that is, the mathematical shape model.

In contrast to the present study, their investigation was based on this line and did not consider the areal extent of the implant. However, it is striking that both independent investigations lead to several similar results and conclusions:

1. Comparison of different CA electrode arrays results in large variations. For optimized insertion, average values are less useful. Instead, the specific curling behaviour should be taken into consideration.
2. Comparison of the curling behaviour of the same CA electrode array after repeated measurements produces fairly repeatable results.
3. The shape of the CA electrode arrays does not fit the cochlear models employed. Instead, interference with the anatomical boundary can be observed. Therefore, a purely kinematic model cannot visualize the complex interaction as is possible with FEA.
4. The optimization of controlled insertion of a movable electrode array is more effective if the array's orientation with respect to the cochlea can be changed.

In the context of their research on robot assistance for CI electrode insertion, Pile et al. [11] studied optimization of insertion by adjusting the orientation and location of the electrode array with respect to the cochlea. This means that their optimization algorithm covered four degrees of freedom, of which three correspond to the parameters Δ*x* and Δ*y* and the orientation Δ*φ* as used in this study. The fourth parameter was an additional possible movement in the *x* direction. This investigation was carried out using the measured curling behaviour of a set of seven CA electrodes and a mathematical cochlear model describing an average human scala tympani. Although the simulated robot-assisted insertion process was not compared with a conventional approach, their findings support the idea of automated insertion incorporating the specific “shape kinematic” (equivalent to what is referred to here as “curling behaviour”). The curling behaviour resulting from the Advanced Off-Stylet (AOS) technique was described as “incapable of not interfering to some degree with the inner wall of the scala tympani.” This is in accordance with the finding in this study that there is a fundamental mismatch between implant curling and cochlear shape. While the curling of the electrode array starts at the tip with immediate and full recovery of the final shape due to stylet extraction, the electrode array is at that time located in the basal part of the cochlea, which is only slightly curved compared with the apical shape. Only at the end of the insertion process do the shape of the implant and the shape of the cochlea exhibit their best fit (notwithstanding that the electrode array has an average spiral shape and does not fit the actual individual shape of the inner ear).

### 4.2. Error Analysis: Quality and Reliability of the Modelling

#### 4.2.1. Modelling the Contour Advance Electrode Array

The modelling of the electrode array based on the detected position of the platinum contact electrodes and the manually marked Softip proved to be useful [10]. Threshold-based segmentation algorithms had been tried out but did not provide usable results due to the weak contrast between the transparent silicone body and the background of the images. In the two-dimensional (2D) model, the basal diameter is (at 0.8 mm) as specified by the manufacturer's data sheet. Toward the tip, however, the implant diameter modelled was, at 0.6 mm by 0.1 mm, larger than specified. This was the only way in which, for the vast majority of the images, it was possible to map the outer contour of the electrode array with sufficient accuracy. The reason is that the fitted central path, which represents the location of the platinum contacts and therefore the shape of the implant, tends to be shifted too far to the inside with increasing proximity to the tip. As a consequence, the inner contour is regularly drawn slightly offset from the true inner wall of the electrode array, which had to be compensated for by the larger diameter. However, total deviations are in the range of less than 0.1–0.2 mm. Modelling of the CA electrode array is thus deemed sufficiently accurate.

#### 4.2.2. Modelling the Inner Ear

A similar value is estimated for the modelling error of the inner ear. Modelling quality is directly correlated with the segmentation which is, in turn, directly related to the quality and resolution of the imaging technique. FpVCT, employed in this study, is of high quality (compared with other clinically used X-ray-based modalities) and provides voxels with an isometric size of 0.2 mm. However, this means that a shift of the segmentation boundary by one voxel causes a change in the size of the cochlea model (again, by 0.2 mm). Additionally, there are errors due to the generation mesh, which triangulates the surface of the segmented object, as part of the export into the STL file.

The biggest shortcoming of the inner ear modelling is the absence of soft-tissue information (owing to the radiological imaging method). Therefore, the basilar membrane is not visible and the segmentation is not limited to the (actually crucial) scala tympani. Instead, the complete bony labyrinth of the inner ear is utilized for the measurement as described in Section 2.3.1. As a result, there is overestimation of the cross-sectional size of the scala tympani. The measurement procedure determines the maximum extension of the spiral-shaped cavity in the bony labyrinth approximately at the level of the basilar membrane. Assuming the implant occupies a central position inside the scala tympani, there is less space inside the “real” scala tympani than the cochlear contour projected in SimCInsert suggests. The extent of the associated error directly depends on the quality of the segmentation and the “vertical” location of the electrode array inside the scala tympani and is thus hardly quantifiable. In any case, it is obvious that, with more accurate sizing of the scala tympani, the trauma risk will be rated slightly higher for all the simulated insertions. The comparative evaluation of the different scenarios, however, remains unaffected by this, as do the conclusions drawn.

### 4.3. Discussion of the Trauma Risk-Rating Scale

#### 4.3.1. Pros and Cons of the Subjective Method

The main disadvantage of the simplified modelling approach used in SimCInsert is the lack of computational structural mechanics. Instead of a “physical” simulation, as is possible with finite element analysis (FEA), the superposition of the interacting objects (electrode array and cochlear anatomy) is only visualized. Thus, the deformation of the flexible electrode array as a result of direct contact with the rigid cochlear walls is not calculated and therefore not displayed. Instead, the contact between both objects is visualized only as an intersection of both contours.

At an early stage of the project, an “objective” method of measurement was implemented in SimCInsert by calculating the depth of the intersection of the first contact area as the perpendicular distance between the farthest point of the contour of the electrode array and the cochlear contour. Although this method provided numerical values which were independent of the individual investigator, this approach was rejected because secondary contact of the implant inside the cochlea could not be taken into account. Thus, this measurement method is not suitable for assessing the overall intracochlear situation. Furthermore, numerical values misleadingly suggest accuracy. As there is no calculation of deformation, the correctness and validity of an “objective” measurement method of this nature are just as questionable as the preferred subjective rating method.

On the other hand, it is an advantage of the presented rating scale that users can benefit from their background knowledge about the flexible behaviour of the electrode array, as had been observed during insertion experiments on artificial cochlear models in the past [39]. Thus, the mechanical-deformation properties of the electrode array are taken more fully into account during the assessment of the contour damage than is possible with a strictly mathematical analysis of the simulation. The legitimacy of the subjective rating method is further strengthened by the fact that the introduced trauma risk is an assessment of the potential risk, that is, not a simulation of direct causal relationships. The direct prediction of real intracochlear damage is not possible with the SimCInsert tool because of the high complexity of the implant's interaction with the surrounding tissues. It is, therefore, of minor importance whether the trauma evaluation is carried out by a subjective assessment or an objective measurement. In both cases, the “trauma risk” allows only a qualitative statement about the likelihood (“very low” to “very high”) that inner ear injury will occur.

#### 4.3.2. Trauma Grade versus Trauma Risk

It is important to bear in mind that the introduced “trauma risk” rating scale is usable only for estimating the potential risk, that is, for assessing the risk of insertion trauma in advance (prospective). In contrast to Eshragi's retrospective “trauma grade”, it is not possible to rate or even to predict actual damage to intracochlear structures.

Therefore, it cannot be ruled out that a specific insertion, which is rated in the simulation with a high degree of “trauma risk,” could be completely atraumatic in the (hypothetical) case of an identical real insertion. Conversely, some uncertainty remains concerning whether a simulated insertion rated with a low risk of intracochlear trauma could lead to significant hearing loss, if it has been possible to exactly experimentally reproduce the simulation. Of course, the already-mentioned complexity of the implant-tissue interaction, the necessary simplifications for SimCInsert, and, finally, the fundamental limitations of the available analysis methods and technologies render such an experimental verification of the simulation results effectively impossible. To clarify the distinction regarding the actual trauma grade using Eshragi's rating scale, Roman numerals were used for the “trauma risk.”

### 4.4. Validity of Simulation Results

#### 4.4.1. manAOS

The simulation of the manually performed AOS insertion technique was based on the presumption that there is (intuitive) adjustment of the insertion process by the surgeon. This assumption was derived from observations, both directly in the operating theatre and using surgical videos. A compensation movement can be regularly observed, which is used by the surgeon to overcome the slight initial curvature of the electrode array when passing it through the incised round window membrane or the cochleostomy. Imperfect straightening of this kind can be found for all known perimodiolar electrode arrays and for certain straight ones (e.g., the Hybrid-L electrode, Cochlear Ltd.). Due to its extent (up to several millimetres), this movement is of relevance for the insertion process. After projection of the movement into the 2D simulation environment SimCInsert, it is equivalent to pivoting around the cochleostomy (Δ*φ*). Taking this compensation movement into consideration for the modelling of the manual procedure is therefore justified and necessary if realistic results are to be obtained. In consequence, it was supposed that an ideal straight-line insertion (i.e., without rotational and translational motions of the implant which superimposes the feed) does not correspond to reality. The quantitative extent of this compensation movement has not, however, yet been measured and is, hence, an aspect that is potentially controversial. However, feasible measurement technologies that address this issue (e.g., stereooptical navigation systems) need to be very accurate, as well as small, lightweight, and inconspicuous, in order not to affect the surgeon's intuitive process. Thus, such technologies are currently not available.

#### 4.4.2. autoAOS

The modelling of the AOS insertion technique as an automated process via minimally invasive access involved greater limitations. Here, the limits of the SimCInsert simulation tool were most clearly noticeable. A straightening effect of the surrounding guiding tube of an insertion tool (or, at least, of the bony wall of the drill canal) on the initial curved electrode array was not observed. Such an effect could only be (highly restrictively and indirectly) incorporated over the course of trauma risk assessment. In contrast, adequate consideration of a guiding tube in SimCInsert is possible only if separate series of measurements are available using mechanical guidance bars to limit the curling behaviour of the investigated electrode arrays during the measuring process. Alternatively, FE analysis is conceivable, which allows appropriate modelling of the restricted guidance of the tube (provided that reasonably accurate knowledge of the material parameters is available).

Of all three examined scenarios, the simulation of autoAOS represents the most consistent implementation of the AOS technique. Since stylet extraction and implant feed were the only two adjustable parameters (but directly linked by indirect proportionality), this is very much in accordance with the manufacturer's recommendations. It also corresponds to the situation when using an automated insertion tool and programs it according to the AOS standard. The integrated actuators will indeed implement the desired movements of implant and stylet in a precise but consistent manner. The insufficient initial straightening of the electrode array, combined with the lack of possibility of performing a compensation movement (owing to the absence of sensory feedback from an open, i.e., not closed, loop control), leads to a high frequency of ratings with grade III or even IV.

Individual results using autoAOS show the advantage of a straight starting configuration. The best outcomes were obtained with electrode array RE07. With the other electrode arrays, however, there are also phases of the insertion process without risk for hearing preservation (grades 0 and I). RE07 is characterized by the flattest curling profile and shows pronounced straightening in the initial phase. The shape of the “trauma risk” curve during insertion indicates that the process is less traumatic in the basal part of the cochlea than in the apical region (see Figure 13). These simulation-based results are confirmed by insertion experiments on transparent artificial cochlear models [39]. They support the conclusion that initial contact between the electrode array and the cochlear wall in the basal region can be prevented by a straight starting configuration. However, slightly bent electrode arrays touch the inner wall.

In practice, a major deficiency of preformed electrode arrays, namely, the insufficient initial straightening, can be compensated for by a guiding tube. This kind of mechanical straightening of the implants would have an impact on the intracochlear situation in the simulation with SimCInsert comparable to that of the adjustment of the intracochlear position of the electrode array toward a contactless midscala position using Δ*x* and Δ*φ* for manAOS. It is therefore expected that, in practical implementation of autoAOS incorporating a guiding tube, the trauma risk for an insertion process of this kind (referred to as “minAOS”) is in the same range as manAOS.

### 4.5. Individually Optimized Insertion: A Successful Approach

#### 4.5.1. Benefit of Individually Optimized Insertion

**Figure 16:**
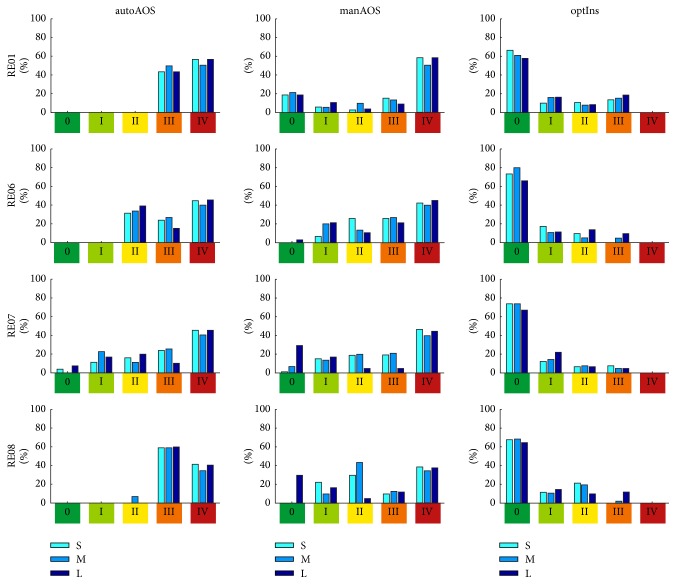
Comparison of results with respect to the electrode array investigated (rows of tabularly arranged histograms). Different cochlear size is indicated by different colours (cf. Figure 5) in the grouped bars of each plot.

**Figure 17:**
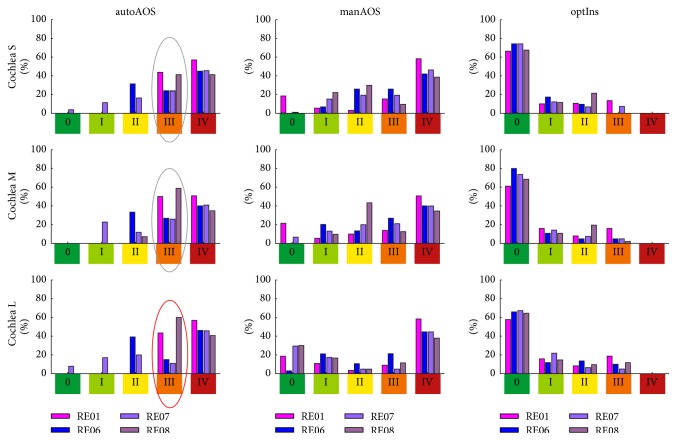
As Figure 16, but now the results of different electrode arrays are shown in grouped bar plots (cf. Figure 3(b)). This means visualizing the results highlights the fact that the influence of cochlear size on trauma risk is less than the actual curling behaviour of the implant. This becomes clear if one compares, for example, the probability of trauma grade III in autoAOS for all three different cochleae. The grouped bar plots are quite similar in appearance (elliptical label), implying that there is not much difference in trauma risk between a small or a medium-sized cochlea. By contrast, in each ellipse, the results for different electrode arrays vary strongly, indicating a strong influence of electrode curling behaviour on the degree of exposure involved in the insertion process.

Notwithstanding the inaccuracies involved in modelling of minAOS/autoAOS due to the insufficient consideration of the influence of a guiding tube, the results presented clearly demonstrate the advantages of individual optimization of the insertion process. Comparison of Figures 12, 10, and 14 clearly indicates that the entire insertion process is shifted toward a gentler procedure, which means a less traumatic and therefore less risky insertion process in terms of hearing preservation. This finding is highlighted by the contrasting juxtaposition, in Figures 16 and 17, of all three insertion strategies investigated. For the first strategy mentioned, the results of the risk rating for the different electrode arrays are shown in separate rows. The outcomes for the small, medium-sized, and large cochleae are summarized within each bar chart. In Figure 17, however, the results are sorted by size of cochlea, with separate rows for CS, CM, and CL. The different electrode arrays are colour-coded in each bar chart. The shift in trauma risk toward lower values due to the increasing level of optimization is clearly visible in Figures 16 and 17: from autoAOS without adjustable insertion parameters, to manAOS with slightly adjustable orientation, and finally optIns representing holistic optimization.

It is noteworthy that the incidence of trauma risk grade IV is nearly equal in those cases with consistent (autoAOS) implementation and manual (manAOS) implementation of the AOS technique. This applies irrespectively of which electrode array (variations in curling behaviour) or which investigated cochlea (anatomical variations) is used. The “intuitively optimized,” manual-insertion-only phases of the insertion process with grade ≤ III can be carried out in a more minimally traumatic manner. Only by considering information about both the individual anatomy and the specific curling behaviour of the inserted electrode array, as was carried out for optIns, is it possible to substantially reduce the risk of insertion trauma.

This remarkable improvement in insertion behaviour with optIns is evident in the high percentage of grade 0 outcomes. For optIns, this degree of risk rating accounts, in all cases, for the largest portion of the total insertion process. After optimization, by adjusting the specific intracochlear curling behaviour of the electrode array to the individual anatomical constraints, the insertion process is unquestionably less risky than without optimization. The working hypothesis is thus substantiated.

The summary of the results in Figures 16 and 17 highlights that the differences in the rating of trauma risk for the insertion of the same electrode array into different cochlea are comparatively low, whereas the outcomes for different arrays but the same cochlea vary far more. In Figure 17, the probability of trauma risk III in the case of an automated AOS insertion is illustrated. The marked grouped bar plots appear quite similar for all three cochleae, indicating that there is not much difference in trauma risk for different-sized cochleae. However, in each bar plot the results for different electrode arrays vary greatly. This finding is a strong indication that insertion behaviour (i.e., insertion forces and insertion trauma) is more dependent on the specific implant's curling characteristics than on the patient-specific anatomy of the inner ear. Hence, the variability of the curling behaviour has a higher impact on the achievable reduction of trauma risk within the optimization process than individual differences do in cochlear anatomy.

**Figure 18:**
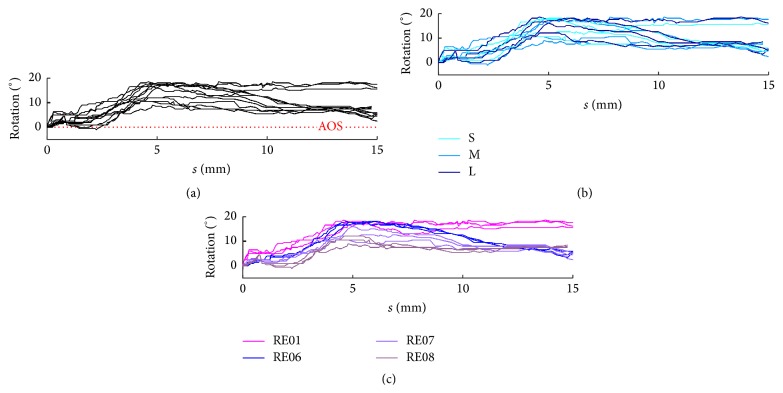
Process parameter Δ*φ* against stylet extraction *s* for all manually optimized insertions, normalized by the initial value at *s* = 0 mm. (a) All 12 optimized simulations compared with the AOS technique without rotation. (b) Different cochlear size is indicated by different colours. (c) Different electrode arrays are colour-coded.

**Figure 19:**
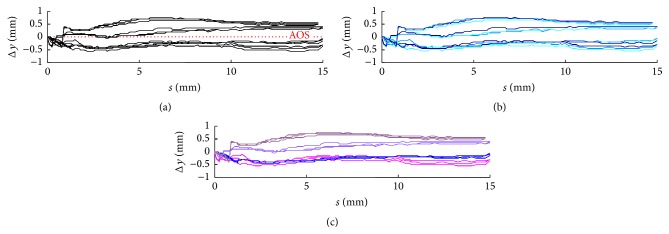
Process parameter Δ*y* against stylet extraction *s* for all manually optimized insertions, normalized by the initial value at *s* = 0 mm. Refer to Figure 18 for legend.

These findings are supported by a closer look at the insertion parameters, as provided in Figures 18 and 19. In both cases, subfigure (a) shows the optimized insertion parameters Δ*φ* and Δ*y* in comparison with the AOS technique. In subfigure (b), the same graphs are colour-coded by cochlear size, and in subfigure (c) equal colors indicate the same electrode array. Only in the second case can regularities be found in the insertion process supporting the conclusion of a greater impact of curling behaviour on the quality of the insertion process. In a converse consideration, this means that only if the curling behaviour of the used implant is known during preoperative planning can the insertion process be significantly optimized. Knowledge only of the individual anatomy (shape of the cochlea) does not allow prediction of the most useful insertion parameters for gentle and therefore less traumatic insertion.

The converse implication of this finding is that improvements to the electrode arrays, such as more predictable curling behaviour, a straight starting configuration, or even steerable electrodes for truly controlled insertion, have strong potential to enhance the insertion process. This primary importance of curling behaviour with regard to an optimization process for electrode insertion is an encouraging outcome in terms of clinical implementation. Even with the available electrode arrays, and later with technical advances to the implants, improved insertion is possible. Only when this optimization potential is fully exploited is a detailed consideration of the individual anatomy necessary for further improvements. Thus, innovative, clinically approved, high-resolution imaging methods to acquire detailed anatomical information on the patient are not absolutely essential in the short term. Improvement of the insertion process is already possible even before these become available. This is why optimization strategies regarding cochlear implant (CI) electrode insertion should address the implant's curling behaviour and curling mechanism.

#### 4.5.2. Limitations

The outcomes of the optimization process also show that it was not possible in all cases to find a “contactless” shape and location for the electrode array inside the inner ear. There are two main reasons why individual optimization of the insertion process by using the CA electrode array is of only limited benefit. The first reason is related to the type of passive curling behaviour which can be found in that type of preformed electrode array. In consequence, that portion of the electrode array which is released from the stiffening effect of the stylet reverts immediately and completely into its manufactured shape. Thus, a characteristic aspect of curling behaviour in the CA electrode is the coiling which starts from the tip of the implant. However, this leads to a fundamental mismatch between the changes in the spiral shape of the electrode array and the spiral shape of the inner ear, one which cannot be overcome by the optimization process. While the electrode array is moved in the basal, and therefore only slightly curved, part of the inner ear, the electrode array already shows the final and maximum curvature in the tip region due to the onset of stylet extraction. Only with total insertion depth and in the final position shapes of the electrode array and the inner ear is there an ideal match.

However, this confirmation is also limited, which leads to the second of the above-mentioned reasons: the average spiral shape with which the CA electrode is produced. The shape of the electrode array is neither individually produced, nor is it possible to individualize the curling behaviour itself (as would be possible with an integrated microactuator array, as described by [34]). Instead, adjustment to the patient-specific shape of the inner ear is (as in the present study) indirectly possible only by tailoring the parameters of the insertion process.

### 4.6. Prospects for Clinical Implementation

The benefits of an individually optimized CI electrode insertion process will lead to noticeable and measurable advantages for the patient only if it can be transferred into clinical practice. As well as the challenges involved in enhancing the experimental prototypes of the automated insertion tool [21, 41] into an intraoperatively usable medical device, the use of the CA electrode array in the context of this new approach necessitates that certain issues be addressed. A central aspect is that the specific curling behaviour of the inserted electrode array must be known or calculable during a preoperative planning and optimization procedure. In other words, the designated electrode array, after removal from the sterile packaging, must show exactly the same curling behaviour in practice that was assumed during the virtual simulation process to achieve the best-possible optimization outcome. Every deviation between the simulated and the actual curling profile reduces the benefits of the optimization process.

**Figure 20:**
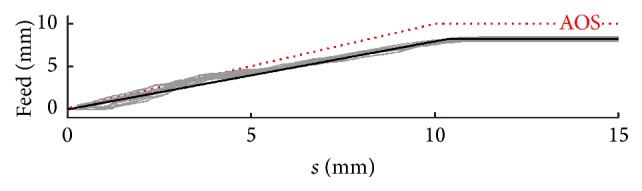
Comparison of the relationship between implant feed (*y* axis) and stylet extraction (*x* axis) for the AOS technique (red dotted line, strong linear relationship as recommended by the manufacturer) and for the optimized insertions (grey lines). The black line indicates an averaged profile for stylet extraction during the insertion process; even this small change appears to be advantageous compared with the conventional AOS technique.

As this entails higher complexity and additional effort, it is appropriate to consider the least time-consuming approach in using average data. This could involve one-off measurements of a representative number of electrode arrays and calculation of an average curling profile. In the course of the preoperative, patient-specific optimization process, this average curling profile needs only to be combined with individual image data for the cochlea. Although both Rau et al. [10] and Pile et al. [11] showed that there is large variability between different CA electrode arrays, a closer look at the results of the present study reveals that even a simplified and limited approach of this nature provides advantages compared with a fully nonoptimized procedure. In particular, for robot-assisted and therefore automated insertion without sensory feedback (and intuitive adjustment, as with the manual approach), anatomy-specific planning would appear to be beneficial, and for several reasons:

1. Although the white marker is a good and established indicator of initial insertion depth (the first step of the AOS technique), this can be improved by taking into account the individual length of the basal turn of the cochlea. As the length between electrode tip and white marker is not dependent on the curling profile, there are no relevant differences between different CA electrodes.
2. A closer look at the relationship between implant feed and stylet extraction shows that there is uniform deviation from the original AOS technique after optimization. As Figure 20 shows, even if an averaged insertion profile (black line) is used, the trauma risk can be reduced. This applies especially to the last two-thirds of the insertion process.
3. Where the AOS technique was employed for the simulation, excessively deep insertion was frequently observed resulting in a large degree of contour damage/intersection. As already mentioned in (2), average limitation of the insertion depth may be expected to reduce insertion forces.

However, the use of the CA electrode array in conjunction average values for curling behaviour has several drawbacks. It should not be forgotten that the CA was not originally designed for this concept. Two independently performed investigations showed high variability in curling behaviour, which limits the usefulness of previously experimentally determined average values. As a consequence, there would be large deviations between the planned and actually performed insertion with corresponding reductions in the benefit from a given optimization. For a comprehensively optimized insertion process, the determination of an averaged curling profile is not an option. In order that the advantages of individually optimized insertion can be fully exploited intraoperatively, it is necessary to compensate for the high amount of variability in curling behaviour. For this purpose, different strategies are under consideration which are listed below (sorted by increasing complexity):

1. Individual determination of the curling behaviour of the electrode array.
2. Design features:
  - Insertion tool with guiding tube.
  - Stiffer stylet for straighter starting configuration.
3. New technologies to reduce variability.
  - Automated manufacturing processes (batch processing) for higher reproducibility of curling behaviour.
  - Active curling mechanism; steerable or controllable curling behaviour.

Clinical implementation in the near future can be achieved by individual determination of curling behaviour. This requires no changes in the design of the electrode array and only minor modifications of the certified manufacturing processes. As regards production, an assembling process already exists by which the stylet is integrated into the electrode array. The measurement can, therefore, be carried out by the CI manufacturer. After reloading of the stylet, the electrode array can be delivered together with the associated curling profile data. This approach is supported by two facts: first, the high degree of reproducibility of the curling behaviour after repeated extraction of the stylet [10, 11] (if the implant is not affected by mechanical forces with plastic deformation) and, secondly, curling behaviour being strongly influenced by the starting configuration and initially exhibiting pronounced variations. After approximately 2 mm of stylet removal, the variation decreases substantially. Relative changes are between 0.1 and 0.2 mm [10] which allows mathematical prediction of subsequent curling. For practical purposes, this means that only the initial phase of curling behaviour needs to be measured, making it significantly easier to determine.

The second strategy to reduce the amount of variability between different electrode arrays is based on the finding that the starting configuration has a strong impact on it. Thus, use of a straightening tube will improve the predictability of curling behaviour and, therefore, the benefit of the optimization procedure. Comparable results could be achieved by use of a stiffer stylet which helps to overcome the slightly curved starting configuration with its (demonstrated and discussed) drawbacks regarding insertion behaviour.

A more forward-looking approach involves research activities in the field of CI electrode development which address how to obtain more controllable and steerable curling behaviour in order to perform a controllable insertion. Different curling mechanism or integrated actuators are currently under investigation, including embedded actuation strands [24, 25], fluidic actuators [29, 30], and the use of smart materials such as Nitinol [31, 32]. In the context of actuated CI electrode arrays, it is worth distinguishing between mechanisms which change curling behaviour in a global manner and those which allow for individual and spatially differentiated changes in implant shape. While the former types of development may provide curling behaviour with higher reproducibility and therefore improved predictability, only the use of multiple microactuators [33, 34], which are arranged inside the electrode array along its longitudinal axis, may allow an insertion which is genuinely individualized. However, this experimental research is at present far removed from clinical practice.

## 5. Conclusion

In this study, it was shown that the passive curling behaviour of clinically established cochlear implant (CI) electrode arrays holds potential for further optimization of the insertion process. By means of controlled, individualized tailoring of the movement and change in shape of the implant to the inner ear anatomy of a given patient, the insertion process can be optimized regarding a reduced risk of intracochlear damage. It is especially noteworthy that this improvement was achieved without any modifications or even development of new, active (i.e., steerable) electrode arrays with an integrated curling mechanism, but rather with a commercially available implant. Therefore, these findings highlight out an entirely novel aspect of clinical application of the Contour Advance (CA) in particular and preformed perimodiolar electrode arrays in general. Although this has not yet been investigated, it is self-evident that comparable improvement can also be achieved using other preformed CIs such as those incorporating MRA or HiFocus Helix electrodes.

## Figures and Tables

**Table 1:** Trauma risk: rating scale for evaluation of trauma risk, ranging from grade 0 to grade IV. Verbal description of the different criteria used for rating each step of electrode insertion.

	Grade of “trauma risk”	Description and representation in the simulation
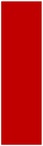	IV	(i) Extensive contour damage, secondary contact areas^†^ due to electrode deformation and restraint^†^ of the electrode array leading to high contact forces (ii) Electrode array is visualized far outside the cochlear contour

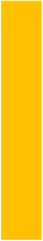	III	(i) Softip intersects with the cochlear contour by more than its total size (ii) Large-scale penetration of the silicone body by more than its total cross section (iii) Pronounced deformation^†^ of the electrode array resulting in secondary contact areas^†^ (iv) Involves additional contour damage^†^, possibly including restraint^†^ of the electrode array between inner and outer walls

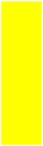	II	(i) Softip intersects with the cochlear contour by more than half its size (ii) Orientation of the Softip contraindicates its yielding with low contact forces (iii) Large-scale penetration of the silicone body by more than a quarter of its cross section

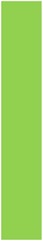	I	(i) Slight contact between the Softip and the inner or outer contour (ii) Slight penetration of the silicone body up to 0.25 of the total cross section (iii) Intersection to such a small extent that it is assumed that the flexible Softip or the elastic electrode array results in (iv) no secondary contact owing to elastic deformations of the implant

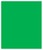	0	(i) No contact between electrode array and cochlea.

^†^Not directly visualized but taken into consideration by the mechanically experienced user.
